# Supplementary material for: Membrane Localized GbTMEM214s Participate in Modulating Cotton Resistance to Verticillium Wilt
Source: Plants (Basel). 2022 Sep 8;11(18):2342. doi: 10.3390/plants11182342 (PMC9505811; doi:10.3390/plants11182342)
Supplement: Supplementary file 1 [file plants-11-02342-s001.zip › plants-1894732-supplementary.pdf]

Table S1: The information of the first three PDB templates with the highest GMQE value

TMEM proteins	Top three templates	GMQE	QMEANDisCo Globa
GbTMEM214-4_D	5f0n.1.A	0.11	0.38 ± 0.06
	6whb.1.A	0.07	0.40 ± 0.07
	6vae.1.A	0.04	0.34 ± 0.08
GbTMEM214-4_A	6whb.1.A	0.08	0.45 ± 0.07
	7lfc.1.A	0.04	0.50 ± 0.10
	6vae.1.A	0.04	0.31 ± 0.08
GbTMEM214-1_D	6whb.1.A	0.09	0.38 ± 0.07
	7p3y.1.B	0.05	0.45 ± 0.09
	5f0o.1.A	0.05	0.37 ± 0.08
GbTMEM214-1_A	3tj1.1.A	0.07	0.37 ± 0.07
	7p3y.1.B	0.05	0.45 ± 0.09
	4ffb.1.C	0.03	0.41 ± 0.11
GbTMEM214-7_D	6epf.1.S	0.04	0.36 ± 0.11
	6f1t.1.4	0.02	0.23 ± 0.12
	1y6x.1.A	0.02	0.42 ± 0.12
GbTMEM214-7_A	6tnf.1.B	0.1	0.31 ± 0.07
	3tj1.1.A	0.08	0.35 ± 0.07
	7p3y.1.B	0.05	0.46 ± 0.09

Table S2: The information of PDB templates for 3D protein modeling

TMEM proteins	PDB template	Description	Sequence Identity	Coverage
GbTMEM214-4_D	5f0n.1.A	cohesin subunit Pds5	15.34%	295-490
GbTMEM214-4_A	6whb.1.A	Mitogen-activated protein kinase kinase kinase 1	13.04%	320-459
GbTMEM214-1_D	6whb.1.A	Mitogen-activated protein kinase kinase kinase 1	11.68%	314-452
GbTMEM214-1_A	3tj1.1.A	RNA polymerase I-specific transcription initiation factor RRN3	10.91%	197-342
GbTMEM214-7_D	6epf.1.S	26S proteasome non-ATPase regulatory subunit 2	13.24%	57-127
GbTMEM214-7_A	6tnf.1.B	Fanconi anemia complementation group I	17.31%	256-439

Table S3 Primer pairs used in this study

Primer pairs	Forward Primer 5'-3'	Reverse Primer 5'-3'	genes
**Primer pairs for gene cloning**			
Primer pairs C1	ATGGAGTCCGTCGATCTTC	TTAGACAGGGATTTGAGAGC	*GbTEME214-1*
Primer pairs C2	CTGTTCTGTTTATTACCCTCC	GAAGCAGGGAAGGTAACTCG	*GbTEME214-4*
Primer pairs C3	CAGTCTTCTCCGACGAAACTCCGT	GGCTGGTGAACCTTGGTCAAGAG	*GbTEME214-7*
**Primer pairs for the quantitative RT-PCR**			
Primer pairs R1	TTTCTCGTTGTCGCATTTGG	AACCGAAGTCTTTAGACAGGGA	*GbTEME214-1*
Primer pairs R2	TCCAAGAAGCAGACAAGT	TTACAACAGCAGCACCTA	*GbTEME214-4*
Primer pairs R3	CACATTTCCAGCACCCTCAG	TTCCAGGAGAACCAGCAAGG	*GbTEME214-7*
**Primer pairs with restriction site for the construction of VIGS vector**			
Primer pairs V1	**GAATTC**CTTACTACTCTTGACCCTTTG	**GGATCC**CCAGATCAAAATGCACTTAACCG	*GbTEME214-1*
Primer pairs V2	**GAATTC**CTACTCTTGATCCTTTGAGG	**GGATCC**GGGGAAGATCGTTAAGAGCT	*GbTEME214-4*
Primer pairs V3	**GAATTC**CAGTGAATGCAGCGCAATTC	**GGATCC**GTGATAGGAAATTTATCCCG	*GbTEME214-7*

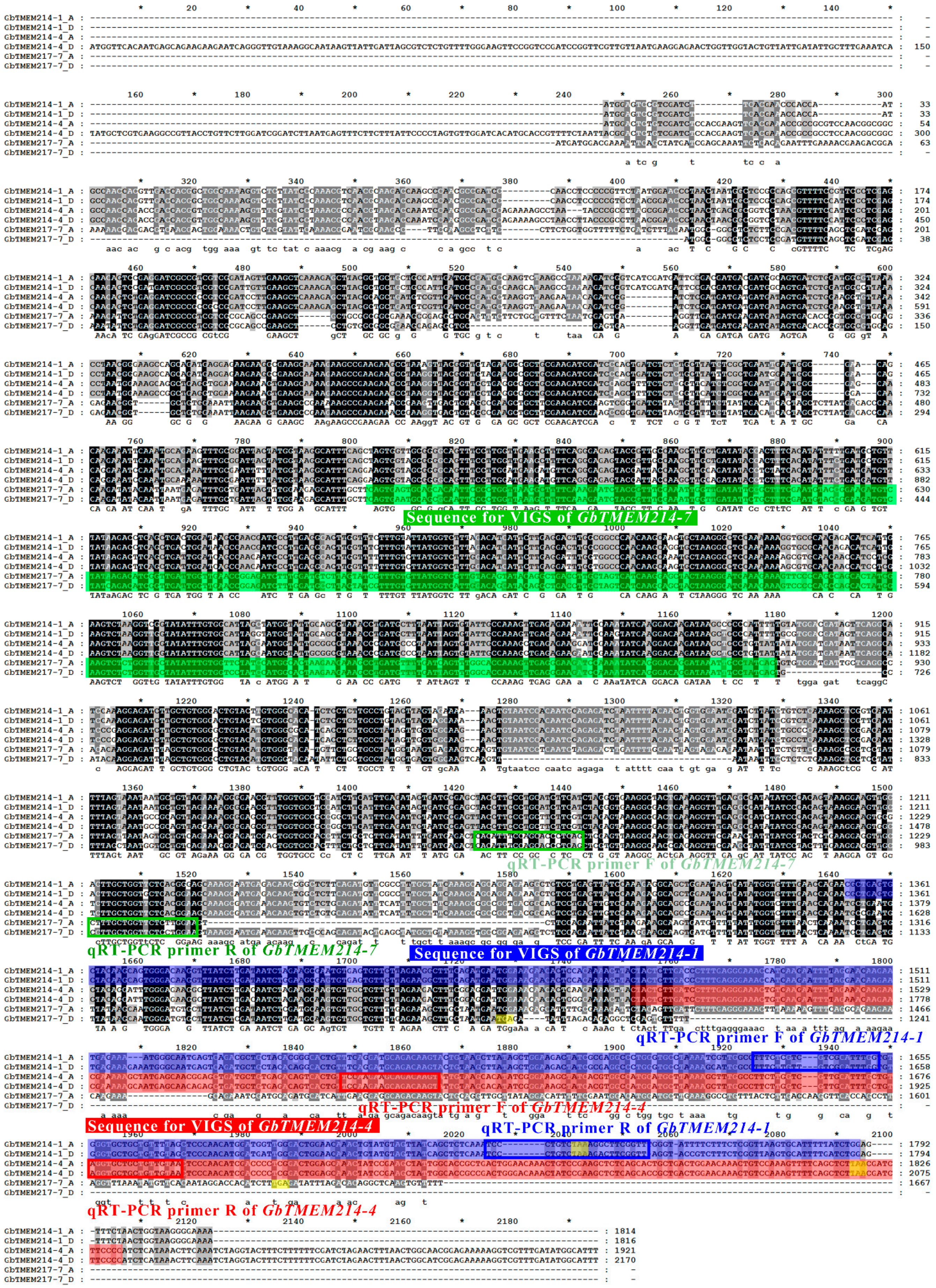

Figure S1. The sequence alignment and primer information of *GbTMEM214s*. The alignment was performed with MEGA 5 software and visualized with GeneDoc software. The sequences for VIGS were marked with shadows of different colors. The primers for qRT-PCR were marked with different color boxes. The termination codons in coding sequences were marked with yellow shadows.

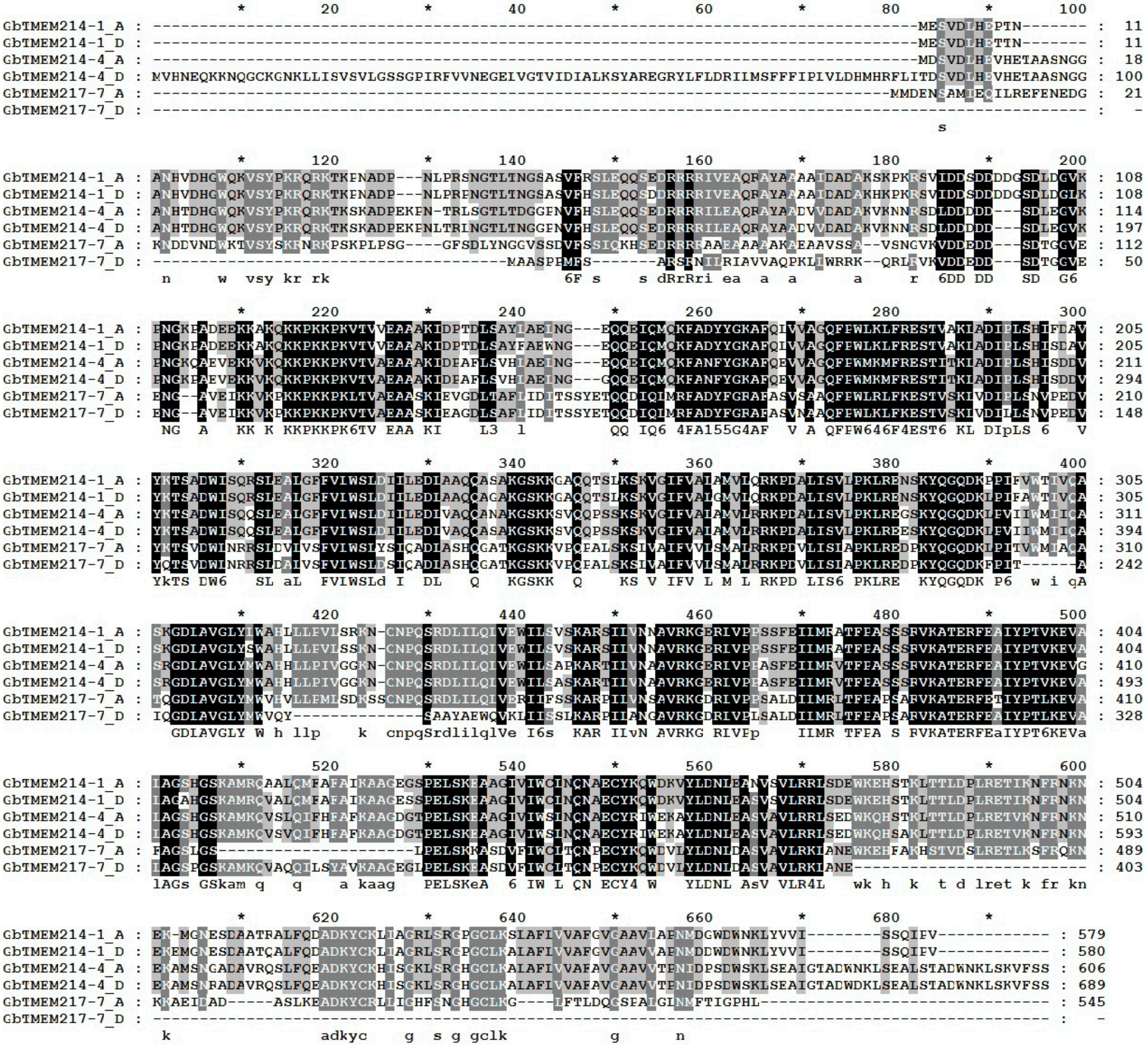

Figure S2. The amino acid sequence alignment of GbTMEM214s. The alignment was performed with MEGA 5 software and visualized with GeneDoc software.

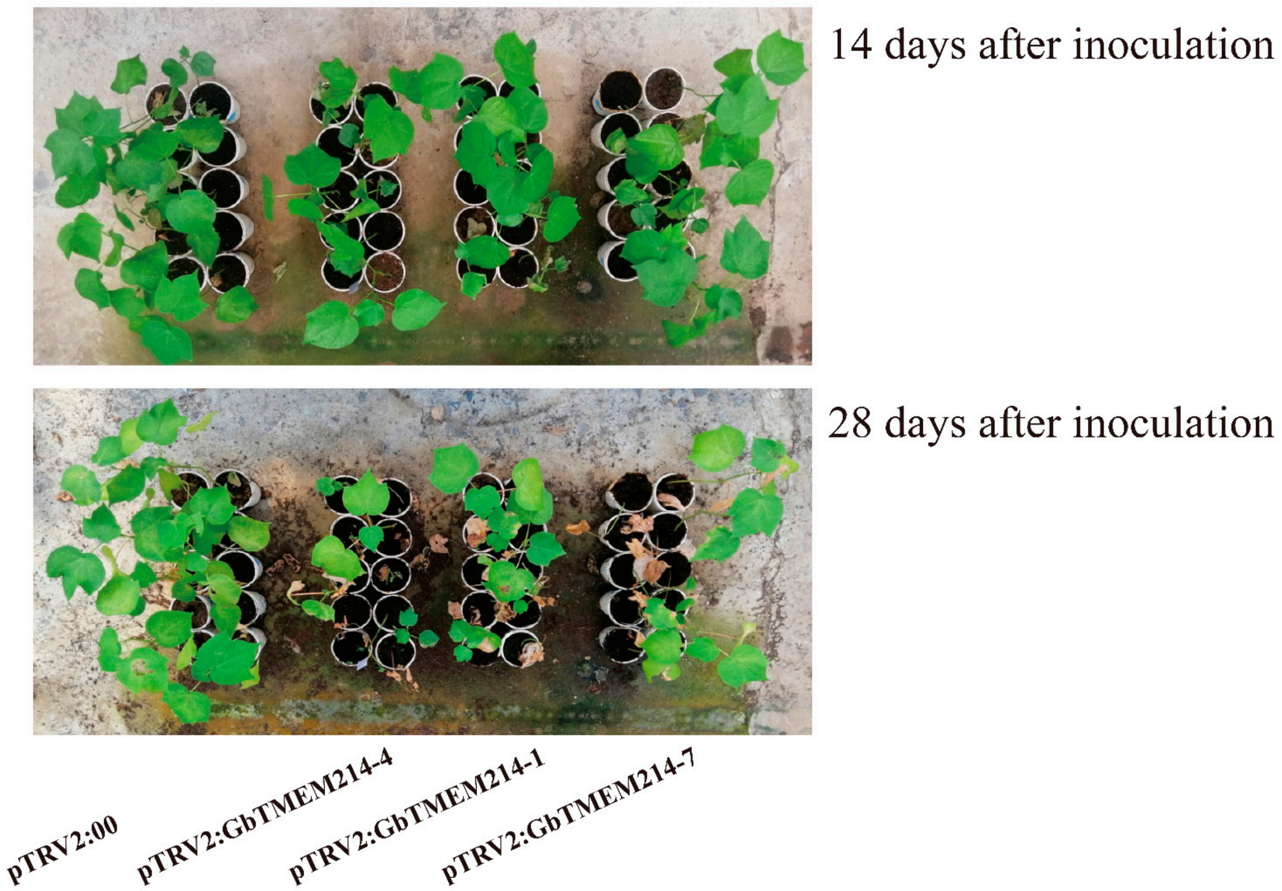

Figure S3. Comparison of phenotypes at different days after inoculation in VIGS analysis.
